# Current status of medication adherence and infant follow up in the prevention of mother to child HIV transmission programme in Addis Ababa: a cohort study

**DOI:** 10.1186/1758-2652-14-50

**Published:** 2011-10-21

**Authors:** Alemnesh H Mirkuzie, Sven Gudmund Hinderaker, Mitike Molla Sisay, Karen Marie Moland, Odd Mørkve

**Affiliations:** 1 Centre for International Health, University of Bergen, OverlegeDanielssens Hus, Årstav. 21, Bergen 5020, Norway; 2 College of Medical and Health Sciences, Department of Nursing and Midwifery, Hawassa University, POBox 1560, Awassa, Ethiopia; 3 School of Public Health, Addis Ababa University, Addis Ababa, Ethiopia; 4 Faculty of Health and Social Sciences, Bergen University College, Department of Nursing, Bergen, Norway

## Abstract

**Background:**

Prevention of mother to child HIV transmission (PMTCT) programmes have great potential to achieve virtual elimination of perinatal HIV transmission provided that PMTCT recommendations are properly followed. This study assessed mothers and infants adherence to medication regimen for PMTCT and the proportions of exposed infants who were followed up in the PMTCT programme.

**Methods:**

A prospective cohort study was conducted among 282 HIV-positive mothers attending 15 health facilities in Addis Ababa, Ethiopia. Descriptive statistics, bivariate and mulitivariate logistic regression analyses were done.

**Results:**

Of 282 mothers enrolled in the cohort, 232 (82%, 95% CI 77-86%) initiated medication during pregnancy, 154 (64%) initiated combined zidovudine (ZDV) prophylaxis regimen while 78 (33%) were initiated lifelong antiretroviral treatment (ART). In total, 171 (60%, 95% CI 55-66%) mothers ingested medication during labour. Of the 221 live born infants (including two sets of twins), 191 (87%, 95% CI 81-90%) ingested ZDV and single-dose nevirapine (sdNVP) at birth. Of the 219 live births (twin births were counted once), 148 (68%, 95% CI 61-73%) mother-infant pairs ingested their medication at birth. Medication ingested by mother-infant pairs at birth was significantly and independently associated with place of delivery. Mother-infant pairs attended in health facilities at birth were more likely (OR 6.7 95% CI 2.90-21.65) to ingest their medication than those who were attended at home. Overall, 189 (86%, 95% CI 80-90%) infants were brought for first pentavalent vaccine and 115 (52%, 95% CI 45-58%) for early infant diagnosis at six-weeks postpartum. Among the infants brought for early diagnosis, 71 (32%, 95% CI 26-39%) had documented HIV test results and six (8.4%) were HIV positive.

**Conclusions:**

We found a progressive decline in medication adherence across the perinatal period. There is a big gap between mediation initiated during pregnancy and actually ingested by the mother-infant pairs at birth. Follow up for HIV-exposed infants seem not to be organized and is inconsistent. In order to maximize effectiveness of the PMTCT programme, the rate of institutional delivery should be increased, the quality of obstetric services should be improved and missed opportunities to exposed infant follow up should be minimized.

## Background

In 2010, the United Nations reported a declining global incidence of HIV among children under the age of 15 years [1]. Most of the decline happened in sub-Saharan Africa where the epidemic is most severe. Among factors that contributed to the decline, prevention of mother to child HIV transmission (PMTCT) programmes were the most significant, according to this report. Currently, a highly efficacious and safe prophylaxis regimen and/or lifelong antiretroviral treatment (ART) that can reduce mother to child transmission (MTCT) to less than 5% are made available in many resource-poor settings [2]. In those settings, however, ensuring uptake and adherence remains a challenge.

Although prophylactic medication coverage during pregnancy has improved significantly from 15% in 2005 to 68% in 2009 in east and southern Africa, it is still lower than the 80% target [1]. Ethiopia is among the worst-performing countries, with less than 20% prophylaxis coverage [3]. There is big gap in initiating medication during pregnancy and mother-infant pairs ingesting the medication at birth, largely due to progressive defaulting that could undermine the efficacy of the medications [4-8]. A low rate of institutional delivery can largely account for a low rate of prophylaxis ingesting at birth by the mother and infants since medications are often available only in health facilities [3,4,9,10].

The other element of PMTCT programmes showing poor uptake and adherence is follow up of exposed infants. Globally, only 15% of HIV-exposed infants access early infant diagnosis [11]. Studies showed that about 20% of HIV-positive infants die before six months and 35% to 40% die before 12 months [11,12]. Early infant diagnosis is a crucial step to facilitate access to ART, to improve infants' survival and to evaluate the effectiveness of PMTCT programmes [11-13].

In the context of PMTCT, most adherence studies from sub-Saharan Africa focus on a single-dose nevirapine regimen while a combined ZDV regimen, despite its complexities and challenges to adherence, has not been well documented. Moreover, there is a scarcity of research in resource-poor settings related to follow up of exposed infants and the rate of MTCT among exposed infants in programmatic settings. This prospective cohort study was conducted in Addis Ababa to assess: 1) adherence to medication regimen among mothers and infants in a PMTCT programme; 2) the proportion of infants followed up in the PMTCT programmes; and 3) the rate of MTCT at six weeks postpartum.

## Methods

The study was conducted in Addis Ababa. The city is the home of about three million culturally and religiously diverse people, and is administratively divided into 10 sub-cities with varying population sizes. In 2009, 54 health facilities were providing integrated perinatal care services, including PMTCT, across the city. Of these, about half were public health centres. Despite the proportional distribution of public and private facilities, the public health centres remained the major providers of perinatal care services, including PMTCT. In public facilities, PMTCT services were provided free of charge. These facilities offered antenatal care to 90% of the pregnant women, conducted 90% of the institutional deliveries and managed 80% of the obstetric complications (unpublished data, collected by Addis Ababa Fistula Hospital, Ethiopian Road Authority and the World Bank), [14]. Of the 54,698 pregnant mothers who attended PMTCT programmes across the city, 79% were tested for HIV and 4.6% were HIV positive [3].

Following HIV-positive diagnoses in antenatal clinics, HIV-positive mothers were referred to ART clinics in order to determine their eligibility either for prophylaxis or lifelong ART. The ART clinics provided several services including: 1) collecting blood samples for CD4 cell and lymphocyte count (the blood sample is then sent to a central laboratory); 2) initiating lifelong ART for mothers with CD4 count of 200 cells/mm^3 ^and less;3) regular monitoring of the mothers' response to ART based on CD4 cell/lymphocyte count; 4) providing adherence counselling using expert patients (HIV-positive volunteer mothers who were trained on adherence); and 5) tracing of ART defaulters (lost to follow up).

Mothers with CD4 counts of 200 cells/mm^3 ^or more initiated combined ZDV prophylaxis in antenatal clinics. The prophylaxis was initiated at 28 weeks of gestation to be taken twice daily and required monthly refill. The antenatal clinics neither provided adherence counsellors nor traced defaulters. Table 1 shows a summary of the prophylaxis regimen recommended in the revised national PMTCT guidelines.

**Table 1:** Medication regimens and infant follow up schedules in the 2007 revised national PMTCT guidelines

*Antepartum*	*Intrapartum*	*Postpartum*
**Mother** If CD4 ≥200 cells/mm^3^; *ZDV from 28 weeks of gestation* If CD4 < 200 cells/mm^3^; *ART*	**Mother** *ZDV + lamuvudine + single-dose nevirapine* *ART* **Infant** *ZDV + single-dose nevirapine*	**Mother** *ZDV + lamuvudine for seven days* *ART* **Infant** *ZDV for 7 days if mother receives the medication ≥ 4 weeks* *ZDV for 1 month if mother receives the medication < 4 weeks*
	**Six-hours follow up** • *Routine early postpartum services for mothers and their infants* • *Infant feeding counselling*	**Six-days follow-up service** • *Routine postpartum care for mothers and infants* • *Infant feeding counselling* **Six-weeks follow up services** • *Routine postpartum care* • *Early infant HIV diagnosis using polymerase chain reaction (PCR) from dried blood spot (DBS)* • *Cotrimoxazole for infants receiving breast milk for opportunistic infections* • *Infant feeding counselling* • *First pentavalent vaccine and other routine child health services*

During the intrapartum period, mothers who initiated prophylaxis were given lamuvudine and sdNVP in addition to ZDV, while those who initiated lifelong ART were required to continue their daily doses. Infants were given ZDV and sdNVP within 72 hours of birth. During the postpartum period, mothers continued taking ZDV for seven days. Infants continued taking ZDV syrup for seven days if their mothers received the medication for one month or more; otherwise the infants took the syrup for one month. Postnatal follow up for exposed infants were recommended at six hours, six days, six weeks, monthly until six months and then every three months until 18 months of age (Table 1). During the six weeks of follow up, the first pentavalent vaccine (haemophilusinfluenzae type B, diphtheria, pertussis, tetanus and hepatitis B) and early infant HIV testing were done. The HIV testing was done in a central laboratory using PCR from DBS, which took a minimum of one month.

The study was a health facility-based prospective cohort conducted from January to December 2009 in 12 public health centres and three private hospitals in Addis Ababa. A four-to-one public-to-private ratio was used in selecting health facilities, considering that more than 80% of the pregnant mothers in the city initiated care from public health facilities. Then individual health facilities were selected on the basis of high client flow and to provide representation of all the 10 sub-cities.

**Figure 1:**
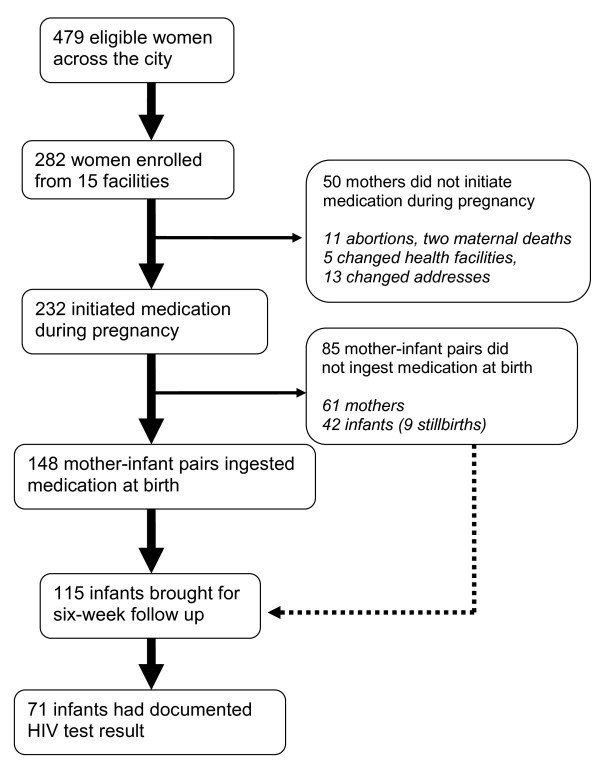
**Study cohort**.

In 2009 alone, 1976 pregnant mothers were diagnosed as HIV positive in PMTCT programmes across the city. Of these, approximately 25% were diagnosed in the first quarter of 2009 (January to March) and were eligible for the study taking into consideration the time required for a baby to be six weeks old by completion of the study. Of the 479 mothers diagnosed from January to March across the city and who were eligible for the study, 282 were attending those health facilities selected for our study, and all consented to be followed up (Figure 1).

The study was reviewed and approved by the Ethical Committee of Addis Ababa City Administration Health Bureau in Ethiopia, as well as the Regional Committee for Medical Research Ethics in Western Norway. Study permits from the Addis Ababa City Administration Health Bureau and the respective sub-cities were obtained.

A semi-structured follow-up format was developed in English for data collection. Most of the variables in the format were obtained from PMTCT and exposed infant logbooks and the national PMTCT guidelines, although some were added from reviewed literatures. The inclusion of routinely recorded variables was considered to minimize incomplete information in the case of loss to follow up; it also ensured data quality as the recruited data collectors had experience in doing routine recording. Thirty-three PMTCT counsellors working in antenatal clinics were recruited to collect the data. They were offered half-day training on the follow-up format and on how to do the follow up. Data were collected anonymously using the women's antenatal numbers as their unique identifiers for ethical reasons.

At enrolment, mothers were interviewed on socio-demographic variables, date of HIV testing and gestational age at enrolment. Follow up data were obtained from the mothers themselves and from logbooks in the facilities. The follow-up schedules of the cohort coincided with the women's regular perinatal visits, i.e., at 28 weeks, 36 weeks, at delivery, six days postpartum and six weeks postpartum. The follow-up data at the 28-week visits were CD4 cell count, whether medication was initiated or not, gestational age medication initiated, type of medication initiated (prophylaxis or lifelong ART), disclosing HIV status to partner, and partner involvement in HIV counselling and testing.

At 36 weeks gestation, adherence to medication was assessed using a one-week recall period. Follow-up data at delivery were place of delivery, mode of delivery, mother prophylaxis during labour, infant sex, infant birth weight, infant status at birth, and infant prophylaxis at birth. For mothers who were transferred to other health facilities and for those who gave birth at home, these data were collected when they came for their six-day and six-week postpartum care visits. The follow-up data at six weeks postpartum were about the type of infant feeding practices, first pentavalent vaccination, whether infants were brought in for early infant diagnosis or not, and whether dried blood spot was taken or not. Infant HIV test results were collected from the facilities a minimum of one month following collections of dried blood spot. Study participants were given a small incentive (about US$2) for transport at each visit.

The main study outcomes were the proportions of: 1) mothers initiating medication during pregnancy; 2) mother-infant pairs ingesting medication at birth; 3) infants brought for early infant diagnosis at six weeks postpartum; and 4) infants tested positive at six weeks postpartum. In this study, the term, "initiate", implies the receiving of medication during pregnancy, and "ingest" implies actual swallowing of the drug observed by health professionals or mothers' self reports at birth. "Adherence" implied documented or self-reported initiating/ingesting of the medications and bringing infants for six-week follow up. Analyzing medication initiated by the mothers during pregnancy, we used abortion and death of a mother as study endpoints. Analyzing medication ingested by mother-infant pairs at birth, we used abortion, death of a mother and stillbirth as study endpoints.

The data were double entered in Microsoft Excel and checked for consistencies and then transferred to SPSS version 17 for analysis. Descriptive statistics, bivariate and multivariate logistic regression analyses were done. Variables with p values of less than 0.2 in bivariate analysis were included in the multivariate model to control for potential confounding effects. A p value < 0.05 was considered significant and a 95% confidence interval (CI) was used. The proportions of mothers who initiated medication during pregnancy were calculated among those who were enrolled into the study. The proportions of mother-infant pairs ingesting medication at birth were calculated from live births, and twin births were counted once. The proportions of infants brought for early infant diagnosis at six weeks were calculated from the total live births. The rate of MTCT was calculated among infants with documented HIV test result.

## Results

The cohort enrolled a total of 282 HIV-positive pregnant women. Of these, 11 mothers had abortions, two were transferred out, and two died (one while pregnant and the other one after birth). In total, 217 mothers had live births of single infants and two mothers had live birth of twins (Figure 1).

Table 2 shows the demographic and obstetric characteristics of the mothers enrolled in the study. The median age of the mothers was 25 years and the median schooling completed was Grade 7. The majority of the mothers were pregnant for the second time. The median gestational age during enrolment was 21 weeks. The mothers had a median CD4 count of 310 cells/mm^3^. In total, 160 (57%) disclosed their HIV-positive status to their partners, 82 partners were involved in HIV counselling and testing, 109 partners reported to be tested, and 75 (69%) partners were HIV positive. By six weeks postpartum, 151 (74%) infants were receiving exclusive breastfeeding, 49 (24%) were receiving exclusive formula feeding, and four (2%) were receiving mixed feeding.

**Table 2:** Socio-demographic and obstetric characteristics of 282 mothers enrolled into the study

Variable	N = 282 n(%)
**Age in years**	
15-24	99(35.6)
25-29	111(39.9)
> 30	68(24.5)
*Median (IQR)*	*25(23-29)*
**Education (grades completed)**	
0-4	87(31.8)
5-8	98(35.8)
≥ 9	89(32.5)
*Median (IQR)*	*7(3-10)*
**Number of pregnancies**	
First	92(33.1)
Second	101(36.3)
Three or more	85(30.6)
*Median (IQR)*	*2(1-3)*
**Gestational age at enrolment**	
< 28 weeks	150(65.8)
≥28 weeks	78(34.2)
*Median (IQR)*	*21(16-28)*
**CD4 cell count/mm^3^**	
< 200	47(26.3)
200-349	55(30.7)
> 349	77(43.0)
*Median (IQR)*	*310(216-433)*
**Disclosed HIV status to partner**	
Yes	160(83.8)
No	31(16.2)
**Partners involved in HIV** **counselling and testing**	
Yes	82(37.6)
No	136(62.4)
**Partners tested for HIV**	
Yes	109(49.8)
No	110(50.2)
**Partners HIV test result**	
Positive	75(68.8)
Negative	34(31.2)

Due to missing values, the numbers may not add up to the total

Of the 282 mothers enrolled, 232 (82%, 95% CI 77-86%) initiated medication during pregnancy. Of these, 154 (64%) initiated ZDV prophylaxis and 78 (33%) lifelong ART, while 50 (17.7%) did not initiate any medication during pregnancy. Among the 50 mothers who did not initiate medication, seven (14%) actively refused the medication prescriptions and 11 (22%) had abortions, five changed health facilities, 13 changed their addresses, two were transferred out, two died, and no reasons were given for 10 mothers. The changes in health facilities and addresses were common among mothers who got to know their HIV status for the first time. These mothers often went to other health facilities to confirm their HIV status and to follow PMTCT programme where they would not be recognized. Two mothers were found registered in two health facilities with the intention of obtaining support from both places.

A total of 109 mothers with documented gestation at medication initiation had come for collecting more of their medication at 36 weeks. These women were assessed for adherence to prescribed medication using a one-week recall period. Adherence to medication was not significantly different between mothers who initiated prophylaxis and those who initiated lifelong ART (p > 0.05). Of the 77 mothers who initiated ZDV prophylaxis, 55 (68%) never missed a dose, 16 (20%) missed one dose, and 10 (12%) missed more than one dose. Of the 28 mothers who initiated ART, 17 (61%) never missed a dose, six (21%) missed one dose and five (18%) missed more than one dose. No significant associations were observed between receiving medication during pregnancy and socio-demographic and obstetric variables, CD4 cell count, gestational age at enrolment, disclosing HIV status to partner, partner's involvement in HIV counselling and testing, partner's HIV testing and partner's HIV test result (p > 0.05).

Out of the 282 enrolled mothers, 171 (60%, 95% CI 55-66%) had ingested medication during labour. Among mothers who initiated medication during pregnancy 26% did not ingest any medication during labour. In total, 228 mothers were reported to have given birth: 211 (92%) did this at health facilities and 17 (8%) at home. Mothers who gave birth at health facilities were more likely to ingest their medication (77%) than mothers who gave birth at home (53%) (OR 2.94, 95% CI 1.08-8.02). Of the 221 infants born alive (including the two sets of twins), 191 (87%, 95% CI 81-90%) ingested medication at birth.

There was a strong association between medication ingested by the mothers and infants at birth and place of delivery. Infants who were delivered at heath facilities were more likely (OR 13.64, 95% CI 4.64-40.12%) to ingest their medication at birth than those who delivered at home. There was no significant association between mother and infant ingesting medication and demographic variables, obstetric variables, CD4 cell count at enrolment, disclosing HIV status to partner and partner being involved in HIV counselling and testing (p > 0.05).

Of the 219 live births (twin births were counted once), 148 (68%, 95% CI 61-73%) mother-infant pairs ingested the medication at birth. Ingesting the medication by the mother-infant pairs was not significantly associated with education, age, number of pregnancies, gestational age at enrolment, CD4 cell count at enrolment, mode of delivery and disclosing HIV status to partner (Table 3). The likelihood of mother-infant pairs ingesting the medication was much higher among those who had facility birth than home birth (OR 6.7, 95% CI 2.90-21.65).

**Table 3:** Bivariate and multivariate associations between mother-infant pairs' non adherence to medication at birth and potential determinants

Variable	Mother-infant pairs ingested medication at birth		Unadjusted OR (95% CI)	Adjusted OR (95% CI)
			
	Yes n(%)	No n(%)		
**Age in years**				
15-24	48(63.2)	28(36.8)	1	
25-29	60(69.8)	26(30.2)	1.35(0.64-2.85)	
≥ 30	37(69.8)	16(30.2)	1.0(0.48-2.11)	
**Education (grade completed)**				
0-4	38(62.3)	23(37.7)	1	
5-8	55(70.5)	23(29.5)	0.69(0.34-1.41)	
≥9	54(73.0)	20(27.0)	0.61(0.30-1.27)	
**Number of pregnancies**				
First	48(69.6)	21(30.4)	1	1
Second	59(73.8)	21(26.3)	0.81(0.40-1.66)	0.60(0.27-1.34)
Three or more	39(58.2)	28(41.8)	1.64(0.81-3.32)	1.34(0.62-2.87)
**Gestational age at enrolment**				
< 28 weeks	78(64.5)	43(35.5)	1	
≥ 28 weeks	47(71.2)	19(28.8)	0.7(0.38-1.40)	
**CD4 cell count/mm^3^**				
≥ 350	49(75.4)	16(24.6)	1	
200-349	31(67.4)	15(32.6)	2.08(0.90-4.80)	
< 200	25(59.5)	17(40.5)	1.48(0.64-3.42)	
**Disclosed to partner**				
Yes	110(71.9)	43(28.1)	1	
No	19(63.3)	11(36.7)	1.48(0.65-3.37)	
**Medication initiated**				
ZDV prophylaxis	101(74.8)	34(25.2)	1	1
Lifelong ART	45(63.4)	26(36.6)	1.72(.92-3.19)	1.85(0.96-3.56)
**Place of delivery**				
Health facility	143(70.8)	59(29.2)	1	1
Home	5(29.4)	12(70.6)	5.82(1.96-17.24)	6.72(2.90-21.65)

Due to missing values, the numbers may not add up to the total

Staff turnover happened in nine of the 15 (60%) study sites, and those counsellors who were initially recruited were replaced by other counsellors. In all, 174 mothers attended facilities that experienced staff turnover, where 49% of the mother-infant pairs did not ingest their medication at birth. Among the 108 mothers who attended those facilities experiencing no staff turnover, 57% of mother-infant pairs had ingested their medication at birth (p > 0.05).

Among the 221 live births, 189 (86%, 95% CI 80-90%) infants were brought for their first pentavalent vaccine (haemophilusinfluenzae type B, diphtheria, pertussis, tetanus and hepatitis B) and 115 (52%, 95% CI 45-58%) for early infant diagnosis at six weeks postpartum. Among the infants brought for early infant diagnosis, only 71 (32%, 95% CI 26-38%) had documented HIV test results and six (8.4%, 95% CI 4-17%) were HIV positive. The major reasons for not having documented HIV test results were: DBS not collected from infants due to lack of trained staff; DBS tests done, but results not collected from central laboratory doing the PCR test; and misplacing of the test results at the facilities. No significant differences were observed among infants receiving different feeding modalities with respect to MTCT. Four infants (8.2%) were HIV positive among 49 infants who received exclusive breastfeeding, two (9.5%) among 21 infants who received exclusive formula feeding, and none among two infants who received mixed feeding.

## Discussion

The PMTCT programme has great potential to achieve virtual elimination of MTCT provided that the recommended interventions are properly followed. Our study showed progressive and marked decline in medication adherence across the perinatal period. Although 82% of the mothers initiated medication during pregnancy, only 68% of the mother-infant pairs ingested the medication at birth. There were unnecessary missed opportunities in exposed infants follow up within the healthcare system. By six weeks postpartum, 86% of the infants received their first pentavalent vaccines, but only 53% were brought for early infant diagnosis. These challenges could seriously undermine the effectiveness of the PMTCT programme, and need thorough consideration.

We found that more than 80% of the mothers initiated medication during pregnancy. This finding compares favourably with several empirical works from Ethiopia and other sub-Sahara African countries [4,9,10,15]. It is also in accordance with the 80% target set by the Joint United Nations Programme on HIV/AIDS and the World Health Organization to offer prophylactic medication for pregnant mothers in order to halve the MTCT by the year 2015 [16]. Although the proportion of mothers who initiated medication during pregnancy reached the 80% target, only two-thirds of the mother-infant pairs had ingested their medication at birth.

In a large-scale cross-sectional study conducted in four African countries, similar gaps are reported. Among 3196 HIV-positive mothers who gave birth, 2278 (71%) initiated nevirapine during pregnancy while only 1725 (54%) mother-infant pairs had actually taken it when checked for cord blood [15]. By contrast, a clinical trial conducted in Zambia and a study from Botswana reported a more than 90% level of adherence to prophylactic medications [10]. These differences could be attributed to better coverage and quality of intrapartum obstetric care services. In Botswana, 97% of pregnant mothers have access to antenatal care and 94% to safe institutional delivery whereas in Addis Ababa, 90% mothers have access to antenatal care, but only 44% have access to safe institutional delivery [17].

In line with this argument, institutional delivery was a significant determinant of mother-infant pairs ingesting medication at birth in our study. It also reflects the situation in Ethiopia where infant prophylaxis is available only at health facilities, and infants delivered at home have less chance to get medications unless they are brought to health facilities by their parents. Other researchers have also reported the place of delivery to be an important and significant predictor of ingesting medication at birth [9,10].

Nevertheless, 23% of the mothers did not ingest any medication at birth despite giving birth at health facilities. This could indicate possible failure within the healthcare system that put the mothers and their infants at increased risk of MTCT. Staff turnover happened in 60% of the antenatal clinics included in our study, and there is little reason to think that the turnover in labour wards is different. There was higher proportion of non-adherence among those who attended facilities that experienced staff turnover than those who did not attend these facilities, although this difference was not statistically significant. High staff turnover and frequent rotation can create a gap filled by less experienced staff with little or no training in PMTCT/ART. Poor knowledge of PMTCT/ART by staff in delivery wards could possibly reduce mothers' and infants' chance of getting appropriate and timely medications.

Contrary to what is reported by Stringer and colleagues, mother-infant pairs' medication adherence was lower among the group that initiated lifelong ART than those who initiated ZDV prophylaxis [15]. At 36 weeks gestation, more mothers who initiated prophylaxis reported that they had never missed medication in the past week compared with those who initiated lifelong ART. This contrasted with what had been observed in most ART clinics, where they were providing adherence counselling using expert clients, regular follow up, and monitoring and tracing of treatment defaulters.

Studies have also shown that adherence counselling and active tracing mechanisms can improve adherence and treatment outcomes, and can also reduce loss to follow up [18,19]. Alternatively, poor adherence among mothers' who initiated lifelong ART could be a reflection of their poor health status. Sick mothers may not able to get to the health facilities to collect medication for themselves, as well as for their infants. As shown in Table 3, among the group who did not adhere to mother-infant pair medication, 40% of the mothers had CD4 counts of 200 cells/mm^3 ^or less. This is consistent with a review and research in Ethiopia where a low CD4 cell count is reported to be a significant marker of poor immune status and disease progression [20,21].

Due to the gaps in receiving medication during pregnancy and actual ingesting of the drug by the mother-infant pairs at birth, PMTCT programme effectiveness could be undermined. Compromised efficacy of prophylaxis regimens are reported when the drugs are taken either by the mothers or their infants only. In a double-blind, randomized, placebo-controlled trial conducted in South Africa, Uganda and Tanzania, the rate of MTCT was 8.9% in the group where mothers and infants initiated the intrapartum and postpartum prophylaxis doses, whereas it was 14.2% in the group where mothers, but not their infants, initiated the intrapartum dose [7,8]. Moreover, the proportion of mothers' receiving medication during pregnancy is a proxy indicator currently used for measuring PMTCT programme success; hence the gaps could threaten the validity of this indictor. Particularly in resource-poor settings where there are marked gaps in antenatal care and institutional delivery coverage, this indicator could overestimate PMTCT programme effectiveness. Careful consideration is required in using "the proportion of mothers' receiving medication during pregnancy" as the indicator of PMTCT programme success in these settings.

Our findings suggest that exposed infant follow up was inconsistent and poorly organized, which could negatively impact the success of the PMTCT programme. The 2007 revised PMTC guidelines clearly stated the need for integrated and comprehensive follow-up services for exposed infants [22]. Despite immunization coverage of more than 80% among the HIV-exposed infants, slightly more than 50% of them attended infant follow up at six weeks and less than one-third had documented HIV test result. This shows lack of integration of HIV care with the under-five child health clinics leading to avoidable missed opportunities.

Consistent with our findings, only 25% of exposed infants were tested for HIV in Mozambique [23]; in Kenya, among 2477 exposed infants, only 40% were tested [5]; and only 59% of babies were tested in Zimbabwe by the age of 15 months [24]. This implies that large missed opportunities are occurring within the health system despite having clear guidelines. In the majority of health facilities, the infant follow-up services were scattered over at least at six service points and were available only two or three days in a week. In a study by Nyandiko and colleagues, the health system was shown to be responsible for the low rate of infant HIV testing [25]. The missed opportunities in infant diagnosis can also delay HIV-positive infants from accessing timely treatment, which is detrimental to their survival [11,13,26]. Therefore, creating integrated strategies to contain the necessary procedures pertinent to exposed infant follow up at one single point within the existing under-five child health clinic could be a way forward for a successful PMTCT programme.

The majority of infants were receiving exclusive breastfeeding and were tested for HIV at six weeks postpartum. The rate of MTCT was 8.4%, which is consistent with the reported rate from a similar study undertaken in Addis Ababa [4]. By contrast, lower rates of MTCT were reported from outside of Ethiopia, 5.7% in the Petra clinical trial and 4.7% in a cohort study for Abidjan among predominantly exclusive breastfed infants tested at six weeks postpartum, but there is an overlap in the confidence intervals [6,7]. The rate of MTCT observed in our study seems to be encouraging compared with the 15-25% MTCT expected at six weeks in the absence of any interventions [27]. Nonetheless, the MTCT reported in our study could be underestimated primarily due to the large proportions of potential non-adherence to medication among the lost-to-follow-up cases.

Secondly, the HIV testing was done at six weeks postpartum and did not account for possible MTCT through breastfeeding. To get further reduction in MTCT among breastfed infants, the 2010 revised guidelines advocates for continuation of mothers' or their infants' medication throughout the breastfeeding period [28]. In this regard, continuous adherence support for extended period can be extremely impotent. This calls for tailored follow-up services integrated into existing maternal and child health programmes with a clear sense of ownership and accountability from staff involved in the care.

One of the limitations of our study is that the rate of MTCT was calculated among infants who completed their follow up to six weeks. This could result in overestimation of the effectiveness the PMTCT programme and also threaten the external validity of the study. Attempts were made to minimize the non-adherence using existing defaulter tracers. Moreover, a qualitative account of mothers' perspectives would have added to the explanation for the loss to follow up. Although the study was conducted as part of a national PMTCT programme and the levels of non-adherence were not different from PMTCT programmes in many sub-Saharan African settings [4,5,23,25,29,30], care should be exercised in generalizing the study findings.

Another limitation is that the internal validity of the study could be affected due to employing several data collectors, but we sought to minimize this risk through the following steps:1) the data collectors were all PMTCT counsellors, who were well acquainted with the issue under study; 2) almost all the variables in the follow-up format were drawn from log books in the facilities used to record routine activities; and 3) all the data collectors were given training.

So far, few cross-sectional studies have reported prophylactic medication coverage in Ethiopia. Our research is the first prospective cohort that has estimated level of non-adherence to PMTCT recommendations and averted infections among HIV-exposed infants. The follow up helped us to identify critical points that many clients are failing in PMTCT programmes.

## Conclusions

Despite the large proportion of mothers initiating medication during pregnancy, the majority of them and their infants did not actually ingest the drugs according to the recommendations at birth. This could challenge the overall effectiveness of the national PMTCT programme. The proportion of mother-infant pairs ingesting medication at birth seems to be a more reliable indicator for PMTCT programme planning and evaluation compared with the proxy indicator currently used (i.e., the proportion of mothers receiving medication during pregnancy). Increasing access to quality intrapartum obstetric care services seems to be fundamental to increasing adherence to the recommended medication at birth to reduce MTCT. The focus should also be on increasing the women's awareness on high level adherence to medication regimen for optimal result and removing barriers to institutional delivery.

Meanwhile, facilitating access to medication in the case of home deliveries should also be focused on. Services pertinent to follow up of exposed infants seem to be inconsistent and undeveloped, and might contribute significantly to avoidable missed opportunities and negatively impact the survival of HIV infected infants. Creating a system to contain the necessary procedures for follow up of exposed infants at a single service point should be considered. Targeted interventions should be developed specifically for HIV-exposed infants within the PMTCT package, which should be integrated within the existing traditional under-five child health services to ensure continuity of care for these children.

## Competing interests

The authors declare that they have no competing interests.

## Authors' contributions

AHM prepared the study proposal, collected and analyzed the data, interpreted the findings and wrote the manuscript. OM was involved in developing the study proposal, supervising the data collection and reviewing the manuscript. SGH was involved in developing the study proposal and reviewing the manuscript. MMS was involved in supervising the data collection and reviewing the manuscript. KMM was involved in developing the study proposal and reviewing the manuscript. All authors have read and approved the final manuscript.
